# Demographic and Socioeconomic Factors as Predictors of Device-Associated Healthcare Infections Before and During the COVID-19 Pandemic

**DOI:** 10.1017/ash.2025.287

**Published:** 2025-09-24

**Authors:** Werner Bischoff, Joni Evans, Catherine Passaretti, Andrea Fernandez

**Affiliations:** 1 Wake Forest University School of Medicine; 2 Wake Forest University School of Medicine; 3 Atrium Health; 4 Wake Forest School of Medicine

## Abstract

**Background:** Social vulnerability factors have been associated with negative health outcomes. However, it remains unclear how they affect device-related infections in different population groups. **Methods:** This retrospective observational cohort study included Central-Line Associated Bloodstream Infections (CLABSI) and Catheter Associated Urinary Tract Infections (CAUTI) in an 850-bed, academic tertiary care facility. Information was collected on patient demographics, the CDC Social Vulnerability Index (SVI), hospitalization, comorbidities, and COVID-19 status. SVI analysis included overall vulnerability comprised of the four themes: socioeconomic status, household characteristics, racial/ethnicity minority status, and housing type/transportation. Chi-square and Wilcoxon rank-sum tests were used for categorical and continuous variable comparisons. GEE models compared pre- and pandemic periods by interrupted time series analysis. **Results:** Between 1/1/2018 to 5/31/2022 98,791 patients were admitted 151,550 times. Of those, 17,796 patients received 29,483 central lines and 45,180 patients had 65,422 Foleys. 314 patients developed 338 CLABSI and 216 patients had 217 CAUTI. 1,552 patients tested positive for COVID-19 with 22 developing CLABSI and 14 CAUTI. The pre-pandemic downward trend in CLABSI and CAUTI was reversed during COVID-19 (p Throughout the study Black patients had higher device days (p In the SVI analysis the socioeconomic theme was associated with higher risk of device-related CLABSI across the entire study (p=0.03). During COVID-19 overall SVI and the household characteristics theme were associated with higher device-related CLABSI rates (p=0.03; p=0.03). Adjusting for race or ethnicity dissolved those associations. For CAUTI race/ethnicity minority status was linked to an event throughout the study (p=0.03). This held true after adjusting for individual race or ethnicity status. No associations were detected in the pre- and pandemic periods for CAUTI. **Conclusions:** Health outcome disparities affected Black (CLABSI and CAUTI) and Hispanic/Latino (CLABSI) patients. Of note, both groups had significantly higher device utilization rates. Per-patient infections increased during the pandemic without altering race/ethnicity differences. Higher race/ethnicity minority status SVI was linked to CAUTI. However, CLABSI were driven by the socioeconomic SVI. The findings can help clarify the relationships between race/ethnicity and other demographic and socioeconomic factors associated with device-related infections on the community and individual level.

